# Management of a massive thoracoabdominal impalement: a case report

**DOI:** 10.1186/1757-7241-17-50

**Published:** 2009-10-07

**Authors:** Chhavi Sawhney, Nita D'souza, Biplab Mishra, Babita Gupta, Subir Das

**Affiliations:** 1 Jay Prakash Narayan Apex Trauma Centre, All India Institute of Medical Sciences, New Delhi-110029, India

## Abstract

A 26 year old male was impaled through his chest and upper abdomen with an iron angle, one and half meter long and five centimeters thick. The iron angle entered the chest, through the epigastrium and exited posteriorly just inferior to the angle of left scapula. The patient was transported to hospital with the iron angle in situ. Positioning the patient for intubation proved a major challenge. An unconventional position for intubation allowed a successful airway management. Paucity of time prevented us from gauging the nature and extent of injury. The challenges posed by massive impalement could be successfully managed due to rapid pre-hospital transfer and co-ordinated team effort.

## Introduction

Thoracoabdominal impalement injuries are relatively uncommon and only a few cases have been reported in the literature [1-5]. A great deal of force is required to impale thorax and thus there is extensive local tissue destruction with elements of both blunt and penetrating injury. Management of such cases provides a challenge to anesthetists and surgeons as the extent of injury is unknown and there is inadequate time for evaluation and resuscitation of the patient. We describe the successful anesthetic management of a major impalement wound of the torso and some principles of management are also highlighted. Written informed consent was obtained from the patient for publication of this case report and any accompanying images.

## Case report

A 26 year old male travelling in a car (front seat) at high speed, crashed into a pile of iron angles beside the road at a construction site. One of the iron angles penetrated the windshield and dash-board of the car and impaled his torso. A welder at the construction site was summoned to cut the iron angle. The impaled segment which was one and half meter in length was left undisturbed. Within 30 minutes of the accident, the patient was shifted to the trauma centre.

On arrival, the patient was conscious, oriented but had excruciating pain. The impaled iron angle was projecting in the anterior-posterior direction. It penetrated the epigastrium and exited from the back at the level of the fifth to eight intercostals space, two and half centimeters from the midline just inferior to the left scapula. The patient had pulse rate of 120 beats per minute, blood pressure 110/60 mmHg and his arterial oxygen saturation was 90%. His breathing was rapid and shallow.

The patient and the iron angle were supported at all times to avoid further manipulation. He was transferred directly to the operating room in the sitting position without any investigations (hematological, biochemistry or radiological).

**Figure 1:**
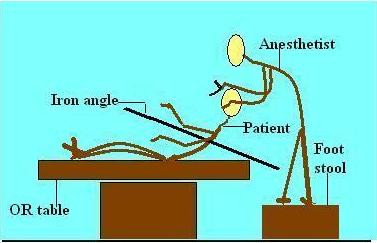
**Position of the patient and the anesthetist during intubation**.

In the operating room he was connected to a multichannel monitor. Two large bore peripheral venous accesses were secured and samples were sent for hematological investigations and blood products were arranged. Even slight change in the patient position or movement of the rod exaggerated the pain so it was decided to intubate the patient in semi-reclining position supported by helpers. The anesthetist stood on the foot stool to gain additional height. After counseling the patient, anesthesia was induced with 2 mg/kg ketamine and 1.5 mg/kg suxamethonium. The trachea was intubated with 8.0 mm cuffed endotracheal tube (Figure 1) under direct vision with minimal external laryngeal manipulation. Bilateral equal air entry was confirmed and the tube position was secured. Right radial artery was cannulated using 20 gauge cannula. The patient was then positioned on the operating table in the right lateral position. Anesthesia was maintained with 60% N20 in oxygen with 0.5-1% isoflurane. Paralysis was maintained with vecuronium and fentanyl 2 mcg/kg was given for analgesia. Intraoperative electrocardiogram, saturation, blood pressure, end-tidal carbon dioxide (ETCO2), temperature and arterial blood gases were continuously monitored. After induction of anesthesia, the protruding portion of the rod was cleaned and wrapped in sterile laparoscopic camera covers so that it could be handled in a sterile manner.

A left thoracoabdominal incision was given joining the entry and exit wounds. The damage incurred was a lacerated left lobe of the liver, transected upper half of the spleen, almost 10 cm rupture of the stomach and divided central tendon of the diaphragm. Posteriorly the diaphragm and the left lung were lacerated before the bar exited the posterior thoracic wall fracturing the 7th, 8th and 9th ribs. The iron angle was lifted under vision directly from the surgical incision. Surgical procedures performed were splenectomy, gastroraphy, left phrenorraphy and the lung laceration was repaired. The surgery lasted for three hours. Total blood loss was approximately 1000 ml. Intraoperative arterial blood gas analysis was within normal limits.

Post operatively, a thoracic epidural was inserted at the level of T **11-12 **space and continuous infusion was started with 0.125% bupivacaine and 2 mcg/kg fentanyl at the rate 4 ml/hour. Patient was shifted to the intensive care unit (ICU) for elective ventilation. The trachea was successfully extubated the next morning. The patient was shifted to the ward after 48 hours and discharged from the hospital after 8 days. No post operative surgical complication, neurological damage or permanent injuries were noted. Presently, the patient is leading a normal life.

## Discussion

Thoracoabdominal impalement is one of the most severe types of penetrating trauma [6]. Such injuries usually involve vital organs, compromising the normal physiology of respiration and circulation. As in any other trauma scenario, there is a trimodal distribution of death. Early deaths occurring within 30 minutes to 3 hours are secondary to hypoxemia, airway obstruction, hemorrhage, haemothorax, cardiac tamponade and aspiration. Complications associated with chest trauma include tracheobronchial tree disruption, diaphragmatic tear, oesophageal disruption, myocardial contusion, pulmonary contusion and thoracic aorta rupture [7].

**Figure 2:**
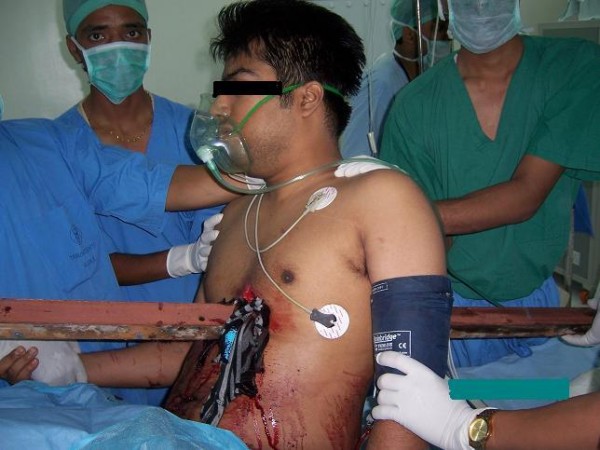
**Patient with the impaled iron angle supported by helpers in the operating room**.

**Figure 3:**
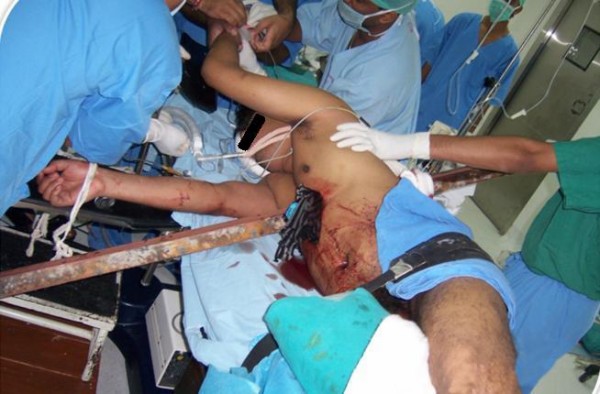
**Post intubation patient given right lateral position for surgery**.

Our patient had penetrating trauma of the left side of the chest which could lead to serious morbidity and mortality in view of the proximity to the vital organs (figures 2 and 3). Patients surviving thoracic impalement injuries are more likely to have sustained injury on the right side, as there is reduced risk of striking the heart or great vessels on this side [8]. In such situations rapid transportation and cautious extrication with minimum manipulation of the impaled object is essential to avoid loss of tamponade effect. Patients who reach the hospital alive generally have high chances of survival. Our patient reached the trauma centre within 30 minutes of the injury with the rod in situ.

In view of multiple life threatening injuries, unknown nature and extent of injury, perioperative management of thoracoabdominal impalement presents a challenge for anesthesia as well as surgery. Reported protocols for management of impalement injury are based entirely on anecdote, still there is uniformity in the suggested approach. Major goals in thoracic trauma resuscitation are optimizing tissue oxygen delivery, control of bleeding and repletion of the intravascular volume. However, care should be taken to avoid over hydration [9].

Most reports suggest that patients in these situations should be rapidly assessed by a targeted examination. Radiographic or other time-consuming investigations should not delay definitive management. In our patient preoperative chest roentgenography (anteroposterior view) was technically impossible due to the protruding rod on both sides of the torso. The information provided by the lateral chest X-ray would have been insufficient to alter the management.

Various positions available for intubation of the patient were considered. One of the options available was to place the patient in lateral position. However, in view of the difficulty in positioning it was decided to intubate him in semi-reclining position. The anesthetist stood on the foot stool to gain additional height. Different authors have described the use of fibreoptic intubation is sitting position [10]. This technique has limited value in emergency situations and may require more time than conventional laryngoscopy. It was not tried in our patient because of paucity of skill as well as due to the urgency for securing the airway [11].

In case of left tracheobronchial injury a double lumen tube would be an ideal option. Due to lack of time and absence of optimal position inserting a double lumen tube could be difficult and lead to airway trauma. Moreover since the patient was maintaining his airway and saturation, it was decided to use a single lumen endotracheal tube

Intravenous induction agents such as propofol and thiopentone may cause a fall in the blood pressure due to vasodilatation and direct myocardial depression. Therefore these should be avoided or administered in titrated doses in hemodynamically unstable patients or patients with suspected cardiovascular compromise. Ketamine is considered useful for inducing anesthesia in patients with cardiac tamponade as it produces sympathetic nervous system stimulation.

Ideally nitrous oxide should be avoided in cases where there is a possibility of closed, unventilated spaces as it makes the patient prone to atelectasis. We instituted nitrous oxide for maintenance of anesthesia after tracheobronchial injury had been ruled out and the extent of injury was ascertained

Adequate pain relief using thoracic epidural catheter helped in early extubation as the patient was able to breathe deeply and cough more effectively. This facilitated co-operation for chest physiotherapy.

To summarize, the outcome after massive thoracic impalement can be improved by [12]:

1. Rapid transportation of the patient

2. Avoiding attempt to remove the impaled object.

3. Targeted examination in hospital without wasting time in unnecessary investigations

A trauma anesthesiologist must be prepared to modify routine techniques in the face of unusual situations. Effective and timely assistance at pre-hospital level with a co-ordinated and trained team work helps in reducing morbidity and mortality of such patients [6,13].

## Consent

Written informed consent was obtained from the patient for publication of this case report and accompanying images. A copy of the written consent is available for review by the Editor-in-Chief of this journal.

## Competing interests

The authors declare that they have no competing interests.

## Authors' contributions

CS, BM and SD conducted and co-ordinated the case. CS, BG and ND conceived the case report, and participated in its design. CS and ND drafted the manuscript and sequence alignment of the report. CS and ND reviewed literature. All authors read and approved the final manuscript.

## References

[B1] Lo Cicero J, Mattox KL (1989). Epidemiology of chest trauma. Surg Clin North Am.

[B2] Mohta M, Kumar P, Mohta A (2006). Experiences with chest trauma: where do we stand today. Indian J Crit Care Med.

[B3] Robicsek F, Daugherty HK, Stansfield AV (1984). Massive chest trauma due to implaement. J Thorac Cardivasc Surg.

[B4] Romero LH, Nagamia HF, Lefemine AA (1978). Massive impalement wound of the chest: a case report. J Thorac Cardivasc Surg.

[B5] Grossi A, Mezzacapo B, Biagi G (1981). Impalement wound of the chest. Thorax.

[B6] Kingsley CI (1999). Perioperative anesthetic management of thoracic trauma. Anesthesiology Clinics of North America.

[B7] Yamamoto L, Schroeder C, Morley D, Beliveau C (2005). Thoracic trauma: the deadly dozen. Crit Care Nurs Q.

[B8] Bowley DM, Gordon MP, Boffard KD (2003). Thoracic impalement after ultralight aircraft crash. J Thorac Cardiovasc Surgery.

[B9] Slinger PD, Campos JH (2009). Anesthesia for thoracic surgery. Miller's Anesthesia.

[B10] Brock-Utne JG, Jaffe RA (1991). Tracheal intubation with patient in sitting position. Brit J Anaesth.

[B11] Snow RG, Nunn JF (1959). Induction of anaesthesia in the foot-down position for patients with a full stomach. Brit J Anaesth.

[B12] Darbhari A, Tandon S, Singh AK (2005). Thoracic impalement injuries. IJTCVS.

[B13] Thomas MO, Ounleye EO (2005). Penetrating Chest Trauma in Nigeria. Asian Cardiovasc Thorac Ann.

